# Anthropogenic Factors Are the Major Cause of Hospital Admission of a Threatened Species, the Grey-Headed Flying Fox (*Pteropus poliocephalus*), in Victoria, Australia

**DOI:** 10.1371/journal.pone.0133638

**Published:** 2015-07-24

**Authors:** Titus Franciscus Scheelings, Sarah Elizabeth Frith

**Affiliations:** 1 Australian Wildlife Health Centre, Healesville Sanctuary, Healesville, Victoria, Australia; 2 The Royal Melbourne Zoological Gardens, Parkville, Victoria, Australia; University of Queensland, AUSTRALIA

## Abstract

To determine the reasons for presentation and outcomes of hospitalised grey-headed flying foxes (*Pteropus poliocephalus*) in Victoria, Australia, a retrospective analysis was performed on 532 records from two wildlife hospitals. Cases were categorised based on presenting signs and outcomes determined. Anthropogenic factors (63.7%) were a major cause of flying fox admissions with entanglement in fruit netting the most significant risk for bats (36.8%). Overall the mortality rate for flying fox admissions was 59.3%. This study highlights the effects of urbanisation on wild animal populations and a need for continued public education in order to reduce morbidity and mortality of wildlife, especially threatened species.

## Introduction

The grey-headed flying fox (*Pteropus poliocephalus*), is one of the largest species of fruit bats and is endemic to the south-east coast of Australia [1]. Its range extends further south than any other species of pteropid bats, with the establishment of colonies in the more southern latitudes likely to have been mediated by anthropogenic climate change [2]. Grey-headed flying foxes roost in large colonies during the day and disperse widely at dusk in search of flowers, fruit and nectar [1,3]. Food sources are often patchily distributed with marked seasonal variability necessitating bats to forage over a wide range of disturbed and intact habitat [1,3]. Flying foxes play an integral role in the Australian ecosystem as pollinators and conduits for seed dispersal [4].

The grey-headed flying fox is listed as Threatened in Victoria, and Vulnerable nationally [5,6]. Key threatening processes that have been identified in declining flying fox populations are predominately anthropogenic in nature and include habitat destruction, roost disturbance and illegal culling by orchardists [7]. Habitat fragmentation, coupled with a combined increase in water and cultivated fruit and flower availability in metropolitan settings, has led to an increase in urbanisation of flying fox colonies and a subsequent increase in human-bat conflict situations [8]. In addition, the recent emergence of a number of significant bat-borne zoonotic diseases in Australia (Lyssavirus, Hendra virus, Menangle virus) [9], has led to a rise in negative public perception regarding flying foxes [8].

Direct and indirect anthropogenic effects have been shown to be an increasing threat to wildlife populations [10–13], and it is important to comprehend the extent of their impact. The purpose of this investigation was to categorise the major reasons for grey-headed flying fox admissions to two veterinary medical centres and to determine the greatest anthropogenic causes of morbidity and mortality in this threatened species.

## Methods

The medical records of 532 grey-headed flying foxes admitted to the Australian Wildlife Health Centre (AWHC) at Healesville Sanctuary (38.68°S 145.53°E) (n = 218) and The Royal Melbourne Zoological Gardens (RMZG) (37.78°S 144.95°E) (n = 314) between January 2000 and December 2014 were examined. Medical records that were incomplete were not included in this investigation. Information that was collected on admission of flying foxes included hospital number, date of admission, location found, reason for presentation, age (adult or juvenile), and in most cases sex of animal. Relevant data was organised into a computerised database (Microsoft Excel 2007, Microsoft Corporation, Redmond, Washington, USA).

Flying foxes were assessed under general anaesthesia by the attending clinician and the diagnoses were classified into categories that included entanglement in fruit netting, barbed wire entanglement, electrical wire entanglement, other entanglement, unknown trauma, gunshot, domestic animal attack (cat or dog), hit by car, heat stress, orphan, sickness, old age, and window strike. All forms of entanglement, domestic animal attack, window strike and hit by car were considered to be indirect anthropogenic causes of case presentation. Where animals had been deliberately targeted by people (such as gunshot), these were considered to be direct anthropogenic presentations. Case outcomes were categorised as alive (released directly by veterinary centre or sent to wildlife volunteer for further rehabilitation), died during course of treatment, euthanized (due to severity of injuries or because animal was involved in bite or scratch of person), or retained as a captive zoo animal. Data was also tested for time of year of presentation as well as year of presentation. A proportion (n = 134) of dead animals were sent to a commercial laboratory for Australian Bat Lyssavirus (ABLV) testing.

Fisher’s exact test was used to test for differences in the proportion of cases in each diagnosis category and to identify gender or age biases. Statistical significance was accepted if *P* ≤ 0.05. For statistical analysis, data was imported into a statistical software program (SYSTAT, Systat Software Inc., Chicago, Illinois 60606, USA).

## Results

Of the 532 flying foxes evaluated, 196 (36.8%) were presented for entanglement in fruit netting. This represented the most significant reason for flying-fox hospital admissions (*P* < 0.0001) (Table 1). Unknown trauma was the second most common primary diagnosis throughout the study period (n = 147, 27.6%). This was followed by barbed wire entanglement (n = 66, 12.4%). In total, 339 (63.7%) of grey-headed flying foxes were presented for known anthropogenic reasons. Three-hundred and thirty-four (98.5%) of these were indirect and 5 (1.5%) were direct (*P* < 0.0001) (Table 1).

**Table 1:** Reasons for presentation of grey-headed flying-foxes (*Pteropus poliocephalus*) to two veterinary medical centres in Melbourne, Australia.

Reason for presentation	Total cases (%)^a^	Alive (%)^b^	Died (%)^b^	Euthanized (%)^b^	Retained captivity (%)^b^	Mortality (%)
Fruit netting entanglement	196 (36.8)	117 (59.7)	10 (5.1)	65 (33.2)	4 (2)	38.3
Unknown Trauma	147 (27.6)	55 (37.4)	20 (13.6)	71 (48.3)	1 (0.1)	61.9
Barbed wire entanglement	66 (12.4)	21 (31.8)	6 (9.1)	37 (56.1)	2 (3)	65.1
Electrical wire	29 (5.5)	3 (10.3)	6 (20.7)	19 (65.5)	1 (3.4)	86.2
Other entanglement	20 (3.7)	13 (65)	1 (5)	6 (30)	0 (0)	35
Sickness	20 (3.7)	7 (35)	2 (10)	11 (55)	0 (0)	65
Vehicle collision	13 (2.4)	2 (15.4)	0 (0)	11 (84.6)	0 (0)	84.6
Orphan	10 (1.9)	9 (90)	0 (0)	0 (0)	1 (10)	0
Heat stress	9 (1.7)	6 (66.7)	2 (22.2)	0 (0)	1 (1.1)	22.2
Domestic animal attack	8 (1.5)	2 (25)	0 (0)	6 (75)	0 (0)	75
Old age	7 (1.3)	0 (0)	0 (0)	7 (100)	0 (0)	100
Gunshot	5 (0.1)	0 (0)	2 (40)	3 (60)	0 (0)	100
Window strike	2 (0.01)	0 (0)	0 (0)	2 (100)	0 (0)	100
**Total**	**532**	**235 (44.2)**	**49 (9.2)**	**238 (44.7)**	**10 (1.9)**	**53.9**

^a^ Percentages are based on total number of bats presented

^b^ Percentages are based on outcomes within subcategories of presentation

**Fig 1:**
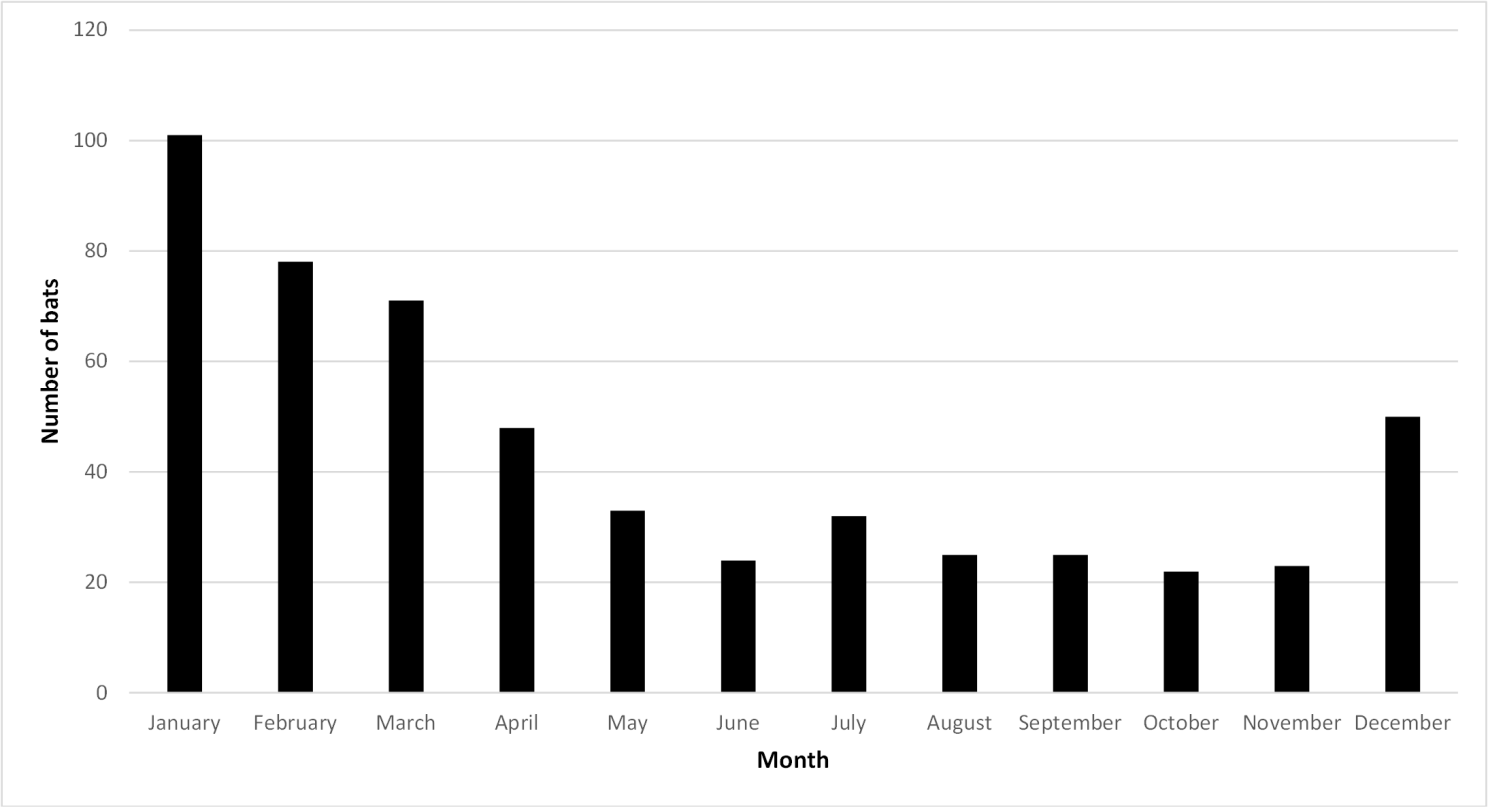
Admissions of grey-headed flying foxes by month. Admissions of grey-headed flying-foxes (*Pteropus poliocephalus*) by month to two veterinary medical centres in Melbourne, Australia.

**Fig 2:**
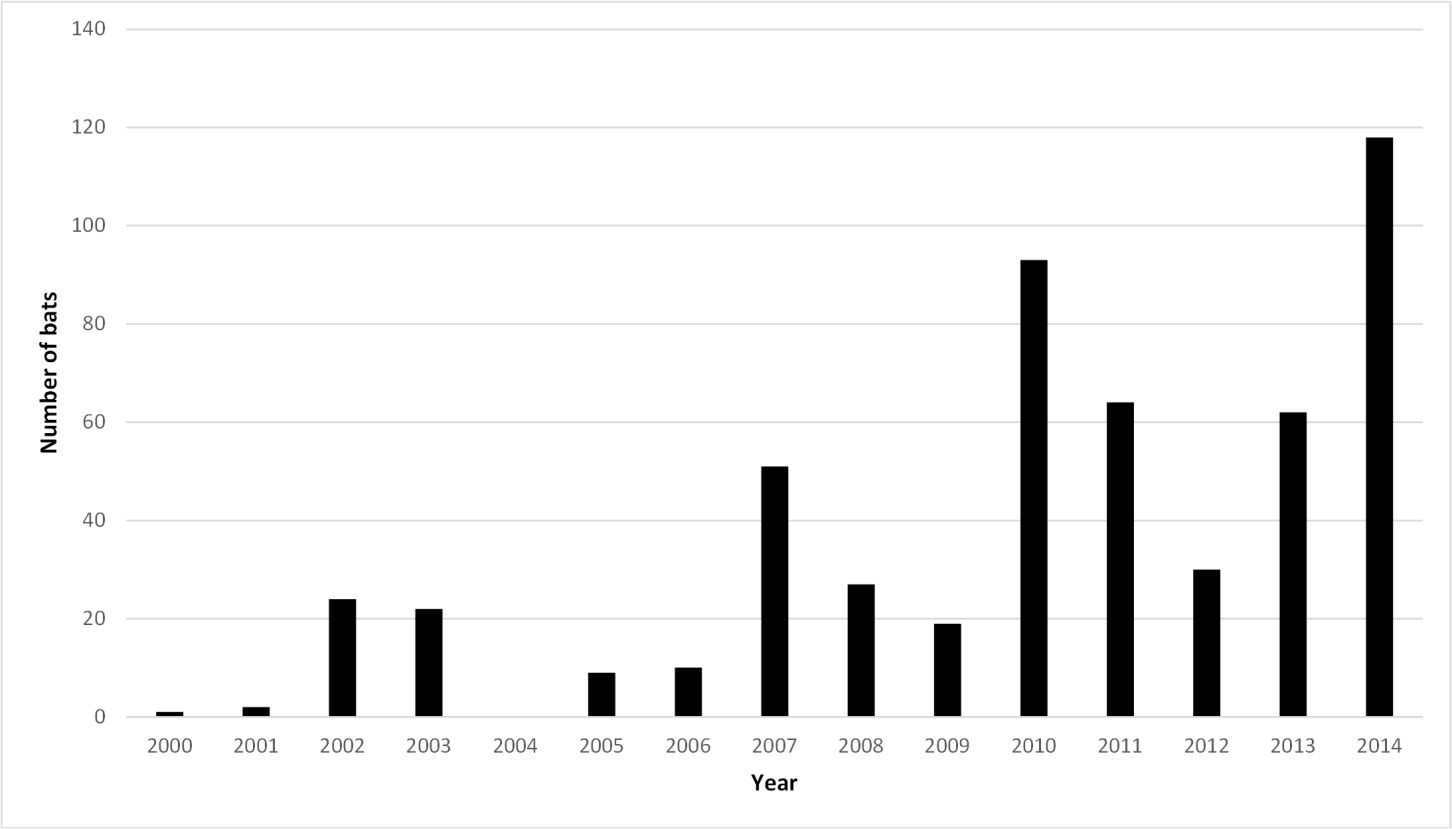
Admissions of grey-headed flying foxes by year. Admissions of grey-headed flying-foxes (*Pteropus poliocephalus*) by year to two veterinary medical centres in Melbourne, Australia.

The sex of grey-headed flying foxes was recorded for 489 (91.9%) individuals. Males (n = 274, 51.5%) were more likely to be presented than females (n = 215, 40.4%) (*P* = 0.0004). The age distribution showed that 82.1% (n = 437) were adult compared with 17.9% (n = 95) which were juvenile (*P* < 0.0001). A significantly higher number of cases were presented in early summer to autumn (December to April) 348 (65.4%), with comparatively few cases admitted during winter or spring (May to November) 174 (32.7%) (*P* < 0.0001) (Fig 1). Peak admissions occurred in 2014 but prior to this there was no obvious trend (Fig 2).

Four case outcomes were identified: released 235 (44.2%), euthanasia 238 (44.7%), died 49 (9.2%), and retained in captivity 10 (1.9%) (Table 1). Presentations with a 100% death rate were gunshot, old age, and window collisions. Other presentations that resulted in a high mortality were vehicle collision, domestic animal attack, and electrical wire. Of the animals that died or were euthanized, 134 (46.7%) were submitted for ABLV testing with an overall prevalence of 4 (3%).

## Discussion

The anthropogenic pressures of an urbanising world place increased demands on wildlife and their habitats. Factors resulting in environmental change include habitat loss or alteration, introduction of invasive species, pathogen spill-over, accumulation of pollutants, and climate change [14]. A sequela of this dramatic environmental manipulation is that wild animals are frequently presented to veterinarians for management of a range of medical conditions, many of which are anthropogenic in nature [10–13]. Therefore, it is incumbent of wildlife veterinarians to be aware of these negative impacts to aid in devising strategies to limit wildlife mortalities [11].

Approximately 64% of grey-headed flying foxes admitted to the wildlife hospitals in this investigation were as a result of anthropogenic factors. Most of these cases were due to indirect contact with humans (entanglement). However, it is possible that this number is an underestimation of the true human impact on flying fox admissions as 27.6% of animals were admitted for unknown trauma. In other similar studies, introduced predators and road trauma were a major reason for wildlife admissions [10–13], but were very rare in this investigation. Flying foxes roost in large colonies, high in trees which affords them a great degree of protection from most dog and cat predators. In addition, they rarely fly close to the ground so are unlikely to be struck by motorists. The major risk factor for urban flying fox populations appears to be entanglement in fruit netting with 36.8% of all cases admitted as a result of this alone. Inappropriate crop protection has previously been identified as a hazard for flying foxes and recommendations for application of wildlife-safe fruit netting have been published [15]. However, this data set suggests that this information has been inadequately disseminated to professional and amateur orchardists and continued community education is required. It is also possible that the current recommended wildlife-safe fruit netting does not prevent entanglement of flying foxes. Further research is required to assess the suitability of specific netting structures as well as alternatives such as scarecrows to scare flying foxes away from orchards.

Grey-headed flying fox admissions were greatest during summer, with peak admissions occurring in January. This is likely due to the increase in fruit availability during this time and also due to an increase in the colony population during the warmer months (both migration and weaning of young) [1,2]. The seasonal increase in flying fox numbers, combined with an overall reduction in native Australian vegetation [16], and a corresponding increase in cultivated fruits, means that bats are forced to rely on human food crops, thus increasing the opportunities for human-bat conflicts [14]. It has been estimated that since European colonization approximately 66% of Victoria’s native vegetation has been cleared making it the most heavily deforested state in Australia [16]. Furthermore, the establishment of a permanent grey-headed flying fox camp in Melbourne is a recent phenomenon and is outside of their historical geographical range [17]. Since 1994, the population at this site has expanded exponentially to a maximum of approximately 30, 000 individuals [17]. This dramatic change in the ecology of pteropid bats has led to closer interactions with humans and domestic animals, resulting in the potential for greater risk of injury to flying foxes and the sporadic emergence of serious zoonotic diseases such as ABLV [10].

This data set suggests an increase in overall flying fox admissions over the study period. Possible explanations for this include a marked increase in the grey-headed flying fox population in Melbourne [17], an increase in availability of cultivated fruits in urban areas resulting in decreased bat migration [18], an increase in public awareness of flying fox conservation, and an increase in recognition of specialty veterinary care required to treat sick or injured bats [McCracken pers comm].

## Conclusion

This investigation examined the major causes of hospital admissions of a threatened species, the grey-headed flying fox. The results of this study indicate that anthropogenic factors, mainly entanglement in fruit netting, are a major cause of hospital admissions of threatened grey-headed flying foxes in Melbourne. The risks of flying fox entanglements are likely to be multifactorial but related to habitat fragmentation leading to urbanisation of bats, and a decrease in native food sources forcing bats to forage on cultivated fruits. Increased public awareness of the dangers associated with inappropriate fruit netting, as well as habitat restoration is required for grey-headed flying fox conservation in Victoria.

## Supporting Information

S1 Appendix Dataset used for analysis in this manuscript. (XLSX)
